# Soil enzyme dynamics under PFAS contamination in arid and Mediterranean soils

**DOI:** 10.1038/s44454-026-00050-4

**Published:** 2026-08-03

**Authors:** Juan C. Sanchez-Hernandez, Natividad I. Navarro Pacheco, Ximena Andrade Cares, Jaroslav Semerád, Tomáš Cajthaml, Mallavarapu Megharaj

**Affiliations:** 1 Laboratory of Ecotoxicology, Institute of Environmental Sciences, University of Castilla-La Mancha, Toledo, Spain; 2 Institute for Environmental Studies, Faculty of Science, Charles University, Prague 2, Czech Republic; 3 Institute of Microbiology of the Czech Academy of Sciences, Prague 4, Czech Republic; 4 Global Centre for Environmental Remediation (GCER), School of Science, College of Engineering, Science and Environment, University of Newcastle, Callaghan, NSW Australia

**Keywords:** Biogeochemistry, Ecology, Ecology, Environmental sciences, Microbiology

## Abstract

Per- and polyfluoroalkyl substances (PFAS) are persistent contaminants increasingly detected in agricultural soils, yet their effects on extracellular enzyme activities remain poorly resolved. This study examined the short- and long-term effects of perfluorooctanoic acid (PFOA) and 6:2 fluorotelomer sulfonic acid (6:2 FTSA) on the biochemical functioning of Mediterranean and arid agricultural soils. Soils were spiked with environmentally relevant concentrations (10, 50, and 250 ng g⁻¹ dry soil) and incubated for 210 days. The tested PFAS concentrations were selected to span literature-reported levels, ranging from typical to highly contaminated conditions. In the arid soil, both PFAS markedly impaired biochemical functioning, with Treated-Soil Quality Index values declining below 70% of control levels. In particular, 6:2 FTSA strongly inhibited β-glucosidase and alkaline phosphomonoesterase activities (up to 81.7%). The Mediterranean soil showed little enzymatic response to PFAS exposure. Eco-enzymatic stoichiometric modeling revealed PFAS-induced shifts in microbial enzyme allocation, particularly in the arid soil after prolonged exposure. PFOA remained stable throughout incubation, whereas 6:2 FTSA dissipated with the formation of perfluoroheptanoic acid. Overall, the arid soil was more biochemically sensitive to both PFAS compounds, suggesting that repeated applications of PFAS-contaminated biosolids or composts pose a greater biochemical risk in dryland agricultural systems.

## Introduction

Sustainable agriculture promotes the use of organic amendments, such as compost and biosolids, to enhance soil fertility, crop yields, and soil organic matter stock^1^. This approach is particularly recommended in regions characterized by low soil organic matter content, including Mediterranean areas and arid regions^2^. However, repeated applications of these materials may lead to the accumulation of persistent contaminants in agricultural soils^3^. Among these, per- and polyfluoroalkyl substances (PFAS) are of increasing concern due to their widespread presence, high persistence, and growing detection in biosolids, urban composts, and soils amended with these organic substrates^4–6^.

PFAS comprise a large and diverse group of synthetic fluorinated compounds that have been extensively used in industrial, commercial, and household products owing to their surfactant properties and high chemical stability^7^. This stability has contributed to their global environmental dispersion, including transport to remote regions^8^. Legacy PFAS, such as perfluorooctanoic acid (PFOA) and perfluorooctane sulfonic acid (PFOS), remain environmentally relevant despite regulatory restrictions on their production and use. Both compounds continue to be detected in sewage sludges, with concentrations ranging from 1 to 5383 ng g⁻¹ for PFOA and from 0.5 to 4780 ng g⁻¹ for PFOS^9^. Consequently, agricultural soils amended with biosolids may contain significant PFAS loads, with concentrations up to 2531 ng g^‒1^ for PFOA and 5500 ng g^‒1^ for PFOS^8^. In response to regulatory measures, the industry has introduced emerging PFAS alternatives with structural modifications, including shorter fluorinated carbon chains, oxygen bridges, or partial substitution of fluorine atoms with hydrogen or other halogens^10^. One such compound is 6:2 fluorotelomer sulfonic acid (6:2 FTSA), a widely used substitute for PFOS that is now also detected in wastewater treatment facilities and biosolids^11,12^. Recent research further indicates that some emerging PFAS can exhibit toxicity comparable to that of legacy PFAS, thus raising concerns regarding their long-term environmental and health impacts^13^.

Research on PFAS in soils has focused mainly on their occurrence, distribution, sorption, mobility, plant uptake, and precursor transformation^14,15^. These investigations have consistently shown that the behavior of PFAS is strongly influenced by soil physicochemical properties, with organic matter often identified as an important driver^16^. This role is supported by experimental evidence indicating that amendments containing sorptive carbonaceous materials, such as biochar, substantially increase the fraction of PFOA that is not easily desorbed in soil, thereby reducing biological uptake^17^. Conversely, soils with low organic matter content tend to exhibit weaker matrix protection and greater microbial inhibition under PFAS stress^16^. Corroborating this pattern, PFOA toxicity in agricultural soil was markedly reduced in fertilized soils with higher organic matter and nutrient availability, indicating that soil characteristics can strongly attenuate biochemical effects^18^. Simultaneously, PFAS are not fully sequestered within the soil matrix; a substantial fraction of PFOA can remain extractable and potentially bioavailable even after aging^19^, while processes involving dissolved organic matter or the rhizosphere may remobilize previously sorbed compounds^20,21^. Collectively, these findings suggest that PFAS may pose a greater, potentially more persistent threat to soil biochemical functioning, particularly in soils with low organic matter than in those rich in organic matter.

Compared with the extensive literature on PFAS fate and transport, there is limited research examining their impact on soil biochemical functions. Some studies indicate that PFAS can alter microbial community structure, respiration, litter decomposition, and selected enzyme activities, but these responses vary with PFAS chemical characteristics and soil properties. For example, PFOS and 6:2 FTSA affected bacterial richness, diversity, and functional capabilities in PFAS-spiked soils, with long-chain PFAS exerting stronger effects than short-chain analogues^22^. Similarly, PFOA altered soil microbial composition and enzymatic activity, with outcomes influenced by the fertilization regime, and with alkaline phosphomonoesterase activity being more sensitive to PFOA exposure than urease activity^18^. Environmentally relevant concentrations of PFOS, PFOA, and perfluorobutanesulfonic acid (PFBS) modified litter decomposition and soil respiration, although most extracellular enzyme activities did not respond significantly under PFAS exposure^23^. More recently, a three-year field study showed that prolonged PFOA exposure altered microbial communities and inhibited alkaline phosphomonoesterase and dehydrogenase activities, whereas urease and catalase were comparatively less responsive, highlighting that enzyme sensitivity is both endpoint-specific and time-dependent^24^. Collectively, these studies suggest that PFAS contamination can disrupt key ecosystem processes linked to carbon and phosphorus cycling.

Despite increasing interest in the ecotoxicological effects of PFAS in soils, most studies have focused on forest^22^ or temperate soils^23^. This leaves a critical gap in understanding PFAS effects in Mediterranean and arid or semiarid agricultural soils. These soils have distinctive physicochemical characteristics, including low organic matter content, high pH and electrical conductivity, which may influence PFAS behavior, bioavailability, and toxicity. Therefore, the main objectives of this study were to: a) evaluate the short- and long-term effects of PFOA and 6:2 FTSA on key soil enzyme activities involved in carbon, nitrogen, and phosphorus cycling, including enzymes closely linked to microbial activity status (dehydrogenase and catalase); b) compare the biochemical response of Mediterranean and arid agricultural soils with contrasting physicochemical properties; and c) assess the persistence of PFOA and 6:2 FTSA in both soil types over time. Based on previous research, we hypothesized that both PFAS would impair soil biochemical functions, with more pronounced and persistent effects in arid soils characterized by extremely low organic matter content and high pH and cation concentrations.

## Results

### Basal enzyme dynamics and carbon and nutrient pools in uncontaminated soils

**Fig. 1:**
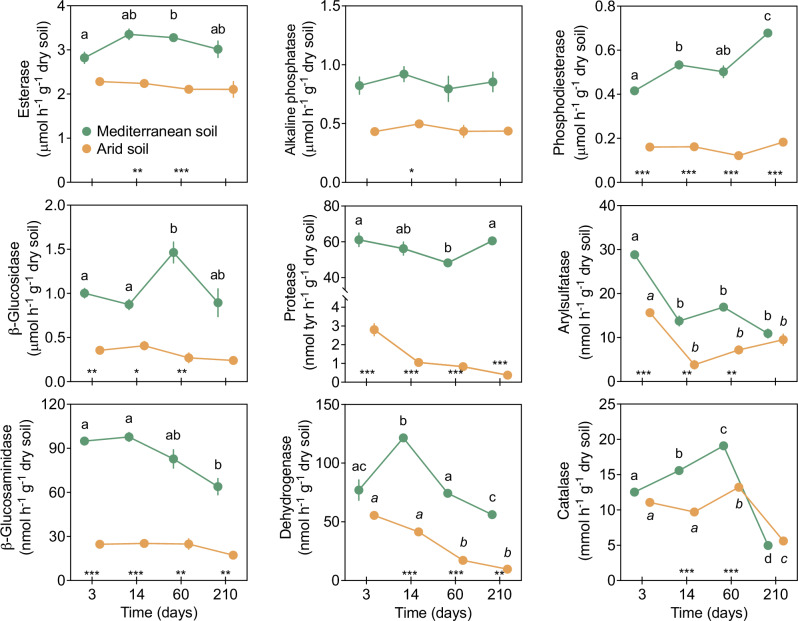
Enzyme activities in control soils. Basal enzyme activities in control, PFAS-free Mediterranean and arid soils during the 210-day incubation period. Values are mean ± SEM, *n* = 4. Different lowercase letters indicate significant differences among sampling times within the same soil according to pairwise comparisons with Holm correction. Asterisks indicate significant differences between soils at a given sampling time (*p < 0.05, **p < 0.01, ***p < 0.001). Two-way RM ANOVA outcomes in Supplementary Table S1.

In control soils, enzyme activities were significantly affected by soil type and incubation time (Fig. 1). Overall, extracellular enzyme activities were lower in the arid soil than in the Mediterranean soil (Supplementary Table S1). In contrast, dehydrogenase and catalase activities did not differ significantly between soils on day 3. Across sampling times, *N*-acetyl-β-D-glucosaminidase (NAG) activity was positively correlated with dehydrogenase activity in both soils (Mediterranean: *r* = 0.60, *p* = 0.017; arid: *r* = 0.60, *p* = 0.016; Supplementary Fig. S1). In the arid soil, dehydrogenase activity was also positively correlated with protease (PRO) activity (*r* = 0.84, *p* < 0.001). Extractable inorganic nutrient concentrations and total organic carbon (TOC) were significantly lower in the arid soil (*P*_i_ = 94.5±4.41 mg kg^−1^ dry soil, NO_3_^−^ = 49.2 ± 9.37 mg kg^−1^, and NH_4_^+^ = 2.41 ± 0.08 mg kg^−1^, TOC = 0.84 ± 0.07%, mean ± SD, *n* = 4) than in the Mediterranean soil (*P*_i_ = 154 ± 2.37 mg kg^−1^ dry soil, NO_3_^−^ = 184 ± 14.0 mg kg^−1^, and NH_4_^+^ = 3.73 ± 0.13 mg kg^−1^, TOC = 4.63 ± 0.47%, mean ± SD, *n* = 4) (Supplementary Fig. S2).

### Effects of PFAS on soil enzyme activities

**Fig. 2:**
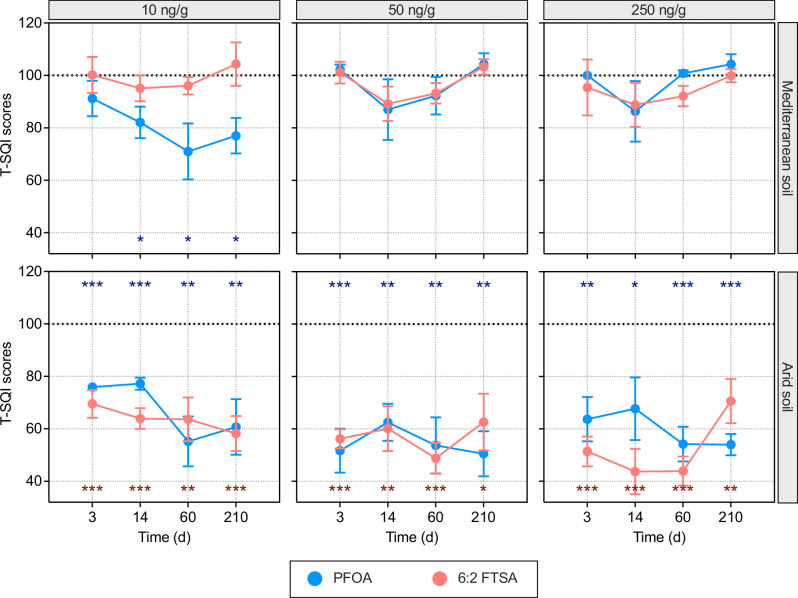
Treated-soil quality index (T-SQI). Time variation of T-SQI in Mediterranean and arid soils spiked with PFOA and 6:2 FTSA. Horizontal dotted lines denote the reference value of control soils set to 100%. *p < 0.05, **p < 0.01, ***p < 0.001 (one-sample *t*-test against control).

**Fig. 3:**
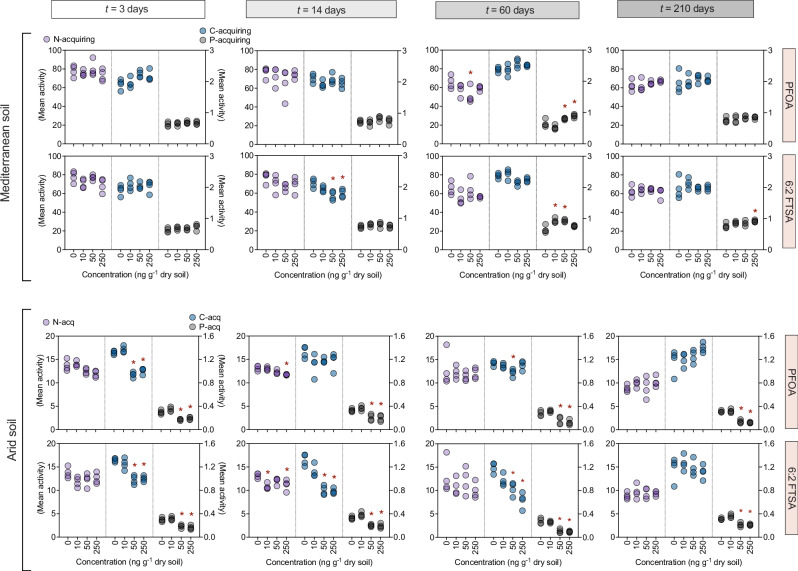
Nutrient-acquiring enzyme categories. Temporal variation of grouped nutrient-acquiring enzyme activities in PFAS-spiked Mediterranean and arid soils. Enzyme activities were grouped into C-acquiring enzymes (esterase and β-glucosidase), N-acquiring enzymes (protease and *N*-acetyl-β-D-glucosaminidase) and P-acquiring enzymes (alkaline phosphomonoesterase and phosphodiesterase). Aligned dot plots show individual replicates (*n* = 4). Differences were evaluated using one-way ANOVA with Welch’s correction followed by Games–Howell post hoc test, *p < 0.05.

Treated-soil quality index (T-SQI) indicated stronger PFAS effects in the arid soil than in the Mediterranean soil, with values falling below 70% of control levels at several sampling times irrespective of PFAS type or concentration (Fig. 2). No clear dose-response relationship was observed across the tested concentration range (10–250 ng g^−1^). In the Mediterranean soil, T-SQI varied little over time or among treatments. Grouping extracellular enzyme activities into nutrient-acquiring categories likewise showed stronger PFAS effects in the arid soil (Fig. 3). Both PFAS inhibited C- and P-acquiring enzymes, with the strongest effects observed for 6:2 FTSA at 50 and 250 ng g^–1^. Relative to controls, esterase (EST) activity decreased by 16.5–49.1%, β-glucosidase (BG) activity by 27.2–73.3%, alkaline phosphomonoesterase (ALP) activity by 40.0–81.7%, and phosphodiesterase (PDE) activity by 15.3–22.5%. Inhibition of P-acquiring enzymes was most pronounced on day 210.

**Fig. 4:**
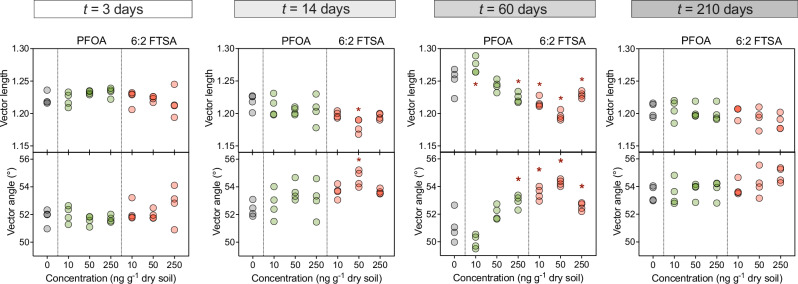
Vector analysis in the Mediterranean soil. Vector length and vector angle values in soils spiked with PFOA and 6:2 FTSA at 0, 10, 50, and 250 ng g^–1^ dry soil. Aligned dot plots show individual replicates (*n* = 4). Differences were evaluated using one-way ANOVA with Welch’s correction followed by Games–Howell post hoc test, *p < 0.05.

**Fig. 5:**
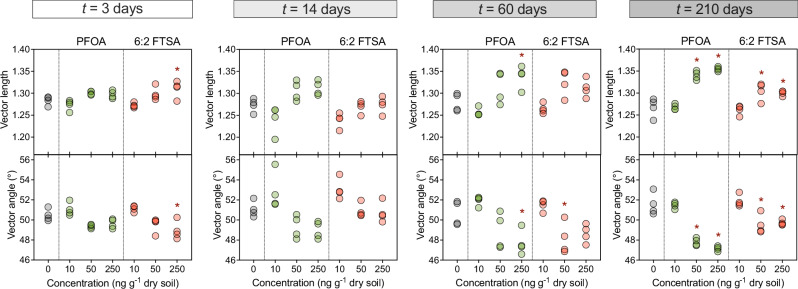
Vector analysis in the arid soil. Vector length and vector angle values in soils spiked with PFOA and 6:2 FTSA at 0, 10, 50, and 250 ng g^–1^ dry soil. Aligned dot plots show individual replicates (*n* = 4). Differences were evaluated using one-way ANOVA with Welch’s correction followed by Games–Howell post hoc test, *p < 0.05.

Vector length and vector angle were used as indicators of apparent microbial resource-acquisition patterns under PFAS exposure. Because these metrics are derived from proportional enzyme activities, they are interpreted here as indicators of apparent nutrient-acquisition constraints rather than direct measures of microbial nutrient limitation. In the Mediterranean soil, vector metrics remained close to control values for most of the incubation period (Fig. 4). The main exception occurred on day 60, when 6:2 FTSA significantly decreased vector length and increased vector angle relative to the control (*p* < 0.05, Dunnett’s post hoc test). In contrast, clearer stoichiometric responses were observed in the arid soil after prolonged incubation (Fig. 5). On day 210, both PFAS compounds significantly decreased vector length relative to the control. Vector angle also decreased at this time point, suggesting a relative increase in N-acquiring compared with P-acquiring enzyme investment.

### PFAS residues in soils

**Fig. 6:**
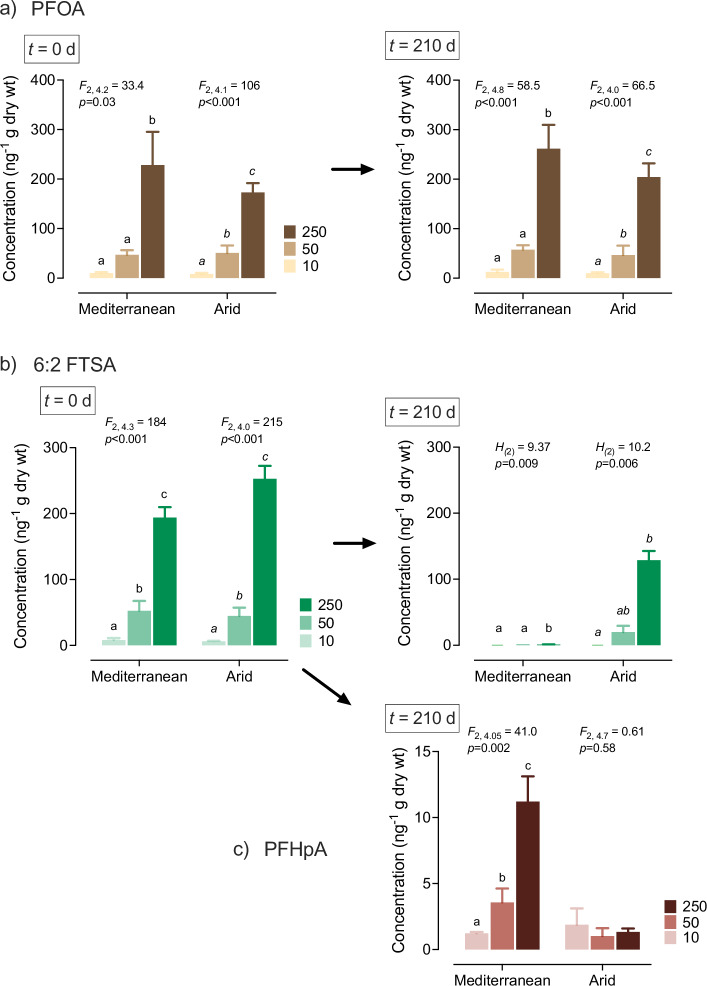
PFAS concentration and dissipation. **a** Measured mean (±SD, *n* = 4) concentrations of PFOA in the Mediterranean and arid soils spiked with nominal concentrations of 10, 50, and 250 ng g^–1^ dry mass at *t* = 0 and after 210 days of soil incubation. **b** Mean (±SD, *n* = 4) concentrations of 6:2 FTSA, and **c** its decomposed terminal PFHpA, after 210 days of incubation. Different letters denote statistical differences between concentrations evaluated by one-way ANOVA with Welch’s correction followed by Games–Howell post hoc test (normal fonts for Mediterranean soil and cursive fonts for arid soil, *p* < 0.05). Significant differences between 6:2 FTSA concentrations for each soil at 210 days were compared by the Kruskal–Wallis test (*H*) followed by Dunn’s post hoc comparisons with Holm correction (*p* < 0.05).

Measured PFOA concentrations in both soils were close to the nominal spiking levels and remained stable throughout the 210-day incubation (Fig. 6a), indicating persistence under the conditions tested. Measured 6:2 FTSA concentrations were also initially close to nominal values in both soils. However, 6:2 FTSA concentrations declined substantially after 210 days, with stronger dissipation in the Mediterranean soil (89.7–100% decrease) than in the arid soil (49.2–100%; Fig. 6b). This decline was accompanied by the appearance of PFHpA (Fig. 6c), whose concentrations were higher in the Mediterranean soil (1.23 ± 0.10 to 11.2 ± 1.90 ng g^–1^ dry soil) than in the arid soil (1.02 ± 0.60 to 1.89 ± 1.23 ng g^–1^ dry soil). Among the 32 targeted PFAS analytes, PFOA and 6:2 FTSA were quantified in accordance with the spiking design, and PFHpA was the only additional analyte monitored that was detected during incubation. The remaining targeted analytes, including screened potential transformation products, were not detected above their respective quantification limits (Supplementary Table S2). PFHpA was not detected above the LOQ in control soils or procedural blanks.

## Discussion

Although extracellular enzyme activities differed between soil types, dehydrogenase and catalase activities did not show significant differences between soils on day 3. This early similarity likely reflected a Birch effect, that is, a transient stimulation of microbial activity following rewetting of previously dry soils^25^. Soil moistening can cause a short-lived flush of available substrates through aggregate disruption and osmotic stress-induced microbial cell lysis, thereby stimulating microbial metabolism^26^. After this initial phase, dehydrogenase and catalase activities declined in both soils, with a stronger decrease in the arid soil. This pattern is consistent with progressive depletion of readily available substrates during incubation. After 210 days, TOC and inorganic nutrient concentrations were substantially lower in the arid soil than in the Mediterranean soil, which likely constrained the maintenance of active microbial biomass over time. By contrast, the higher TOC content of the Mediterranean soil may have contributed to greater temporal stability of microbial activity indicators. The observed correlations between N-acquiring enzyme activities and dehydrogenase or catalase activities are consistent with a microbial contribution to enzyme production during incubation, but they should be interpreted with caution, as correlations do not demonstrate direct microbial control^25^. Extracellular enzymes can persist in soil after release and remain stabilized in organo-mineral complexes independently of viable biomass^27^. Taken together, these results suggest that microbial activity responded strongly to soil rewetting at the start of the incubation and then declined as labile substrates became exhausted, especially in the arid soil. By contrast, most extracellular enzyme activities were comparatively stable over time, likely because part of the extracellular enzyme pool remained protected within the soil organic matrix. This stabilizing effect may have been stronger in the Mediterranean soil, where higher organic matter content could favor enzyme persistence.

We used T-SQI as an integrative metric of changes in soil functioning relative to an unpolluted reference^28^. T-SQI decreased significantly in the arid soil at all PFAS concentrations, indicating that this index is useful for detecting adverse PFAS effects on soil biochemical functioning. Because T-SQI integrates both the magnitude of enzymatic change and the evenness of responses among enzymes, it has previously shown sensitivity to other pollutants, including chlorpyrifos^29^, nanomaterials^30^, and DDT and its metabolites^31^. In our study, PFAS effects were consistently stronger in the arid soil than in the Mediterranean soil. One plausible explanation is that the higher TOC content of the Mediterranean soil promoted stronger PFAS partitioning to the solid phase, thereby reducing the fraction accessible to soil microorganisms^5,32^. This interpretation is also consistent with the lower pH and higher electrical conductivity of the Mediterranean soil relative to the arid soil, because lower pH and higher ionic strength can favor sorption of anionic PFAS. Moreover, pH and electrical conductivity did not vary significantly with PFOA or 6:2 FTSA concentration after 210 days (Supplementary Fig. S3), indicating that these contrasts reflected inherent soil properties rather than PFAS-induced shifts in bulk soil chemistry. However, PFAS bioavailability was not measured directly through pore-water concentrations, extractable fractions, passive sampling, or sorption-desorption analyses. Therefore, the weaker enzymatic response observed in the Mediterranean soil should therefore be interpreted as being consistent with lower expected bioavailability, rather than as direct evidence of reduced PFAS bioavailability.

Among extracellular enzymes, those involved in C and P acquisition were the most sensitive indicators of PFAS exposure, with ALP showing the strongest and most persistent inhibition in the arid soil. This result is consistent with previous studies reporting significant inhibition of ALP activity in PFOA-contaminated soils under different NPK fertilization regimes^18^, and long-term inhibition of ALP and dehydrogenase activity in a three-year PFOA field trial^24^. In both cases, ALP activity was more sensitive to PFOA than enzymes such as urease or catalase. Molecular modeling has further suggested that PFOA may bind directly to ALP through electrostatic interactions, van der Waals forces, and hydrogen bonding^18^. If so, ALP inhibition may partly reflect direct chemical interference rather than only a consequence of reduced microbial activity. Our results are consistent with that interpretation, despite PFAS concentrations that were several orders of magnitude lower than those used in previous studies (10–1000 μg g⁻¹)^18,24^.

Eco-enzymatic stoichiometry provides an integrated framework for evaluating shifts in the relative allocation of microbial enzyme activity to C, N, and P acquisition^33^. This framework has increasingly been used to assess how pollutants alter microbial resource-allocation patterns in soils^33,34^. The strongest evidence comes from metal-contaminated soils, where vector length and angle often change, although the magnitude and direction of those responses depend strongly on soil properties, nutrient status, and contaminant type^35–40^. Similar context dependence has been reported for microplastics^41,42^ and organic pollutants^43,44^, which can alter vector metrics indirectly through changes in carbon availability, aggregation, habitat quality, or microbial stress. In this context, our results suggest that PFAS, particularly 6:2 FTSA, can also modify eco-enzymatic stoichiometry, especially in a low-organic-matter soil with limited buffering capacity. In the arid soil after prolonged incubation, lower vector length and angle were consistent with weaker apparent C limitation and a relative shift toward greater investment in N- versus P-acquiring enzymes. In the Mediterranean soil, by contrast, PFAS effects on vector metrics were small and transient. These shifts should nonetheless be interpreted cautiously, because vector metrics reflect proportional enzyme allocation patterns and do not directly demonstrate changes in microbial growth strategy or realized nutrient limitation.

The dissipation pattern of 6:2 FTSA supports a more complex interpretation than the simple disappearance of the parent compound. Reported transformation products of 6:2 FTSA include short-chain perfluorocarboxylic acids such as PFBA, PFPeA, PFHxA, and PFHpA^45–47^. In proposed aerobic pathways, biotransformation may begin with desulfonation and proceed through intermediates such as the 6:2 fluorotelomer aldehyde (6:2 FTAL) and the 6:2 fluorotelomer alcohol (6:2 FTOH), with PFHpA formed in some systems (Table 1). However, PFHpA is not the dominant end product in many microenvironments, including diluted aerobic sludge^48^, agricultural soils^49^, and bacterial^50–52^ and fungal cultures^53^. By contrast, PFHpA has been reported to account for a fraction of the transformed fluorinated mass in systems such as non-diluted aerobic sludges^54^, hydroponic plant systems^55^, *Rhodococcus* cultures^56,57^, AFFF-impacted soils^58^, aerobic sediments^59^, and landfill leachates^60^. Our results are consistent with this broader pattern. Although PFHpA formation accompanied 6:2 FTSA dissipation, PFHpA accounted for only a small fraction of the lost parent mass. Among the monitored analytes, PFHpA was the only additional compound detected during incubation, whereas the other targeted analytes, including screened potential transformation products such as PFBA, PFPeA, and PFHxA, were not observed above their respective quantification limits. Because our analytical method did not quantify key neutral intermediates such as 6:2 FTAL, 6:2 FTOH, or other non-target transformation products, the fluorinated mass balance remains incomplete. The observed decrease in 6:2 FTSA should therefore be interpreted as evidence consistent with transformation, not as a one-step conversion to PFHpA. This view agrees with previous work showing that parent disappearance and measured terminal PFAS products rarely close the mass balance^58,61^. For example, faster-transforming live soils showed declining total recovery of PFAS, which was attributed to unidentified transformation products^58^. In aerobic soil slurry, more than 90% of the transformed mass remained as intermediate compounds rather than terminal perfluorocarboxylic acids^61^. In addition, volatile intermediates such as 5:2 ketone and 5:2 sFTOH can contribute to apparent mass loss in biotic systems^53^. Therefore, incomplete recovery of fluorinated mass may reflect strong sorption, non-extractable residues, or analytically unresolved products in complex soil matrices^15,19,62^.

**Table 1:** Biotransformation of 6:2 FTSA in different environmental matrices and the main detected intermediates and terminal PFAS products

Incubation medium	Concentration	Stable transformation products	Incubation time	Main findings	Ref.
Diluted activated sludge	1.8–2.6 mg L^–1^	PFPeA, PFHxA, PFBA, 5:3 acid	90 days	6:2 FTS biotransformation relatively slow, with 63.7% of the precursor remaining after 90 d. The microbial aerobic 6:2 FTS desulfonation was the rate-limiting step for further biotransformation. Approximately 24% of the initial dosed 6:2 FTS became irreversibly bound to the activated sludge, making it unavailable for further transformation.	[49]
Bacteria culture (*Gordonia* sp. strain NB4-1Y)	40 μM	6:2 FTCA, 6:2 FTUCA, 5:3 Uacid, 5:3 acid	120 h	Perfluorocarboxylic acids like PFBA and PFHxA were not detected during the 120-h incubation. *Gordonia* sp. strain NB4-1Y used 6:2 FTS as sulfur source for growth. The study identified two nitrilotriacetate monooxygenases likely responsible for initial desulfonation of 6:2 FTS.	[62]
Aerobic and anaerobic systems using river sediments	1.3 mg L^–1^	5:3 acid, PFPeA, PFHA	90 days (aerobic), 100 days (anaerobic)	6:2 FTSA was rapidly biotransformed (half-life <5 days) to stable, terminal products with a total yield of 58 mol% by 90 days. 6:2 FTSA was not significantly breakdown over 100 d; it is likely to persist in anoxic environments.	[60]
Hydroponic culture with pumpkin seedling grown in the solution	500 μg L^–1^ (1100 nmol ml^–1^)	PFHpA, PFBA, PFPrA, TFA, PFHxA, PFPeA	12 days	6:2 FTSA was efficiently taken up by pumpkin roots and translocated to shoots, with higher accumulation in roots than in shoots. PFHpA was the dominant terminal PFAS in roots and PFBA in shoots. Biotransformation is primarily driven by CYP450s, and it involved both α-oxidation and β-oxidation pathways.	[59]
Bacteria culture (*Gordonia* sp. strain NB4-1Y) using sulfur-free mineral medium	60 μM	5:2 FT ketone, 5:2 sFTOH, 6:2 FTOH, 6:2 FTUA, 6:2 FTCA, 5:3 FTCA, PFBA. PFPeA, PFHxA	7 days	The bacterium rapidly degraded (99.9%) 6:2 FTSA by utilizing it as a primary sulfur source. The metabolite 5:3 FT ketone was the dominant product (50% of the total metabolites).	[51]
Wetland slurry (anoxic and aerobic conditions)	200 mg L^–1^	5:3 acid, PFHxA, PFPeA	142 days (aerobic) and 116 (anoxic)	6:2 FTS biotransformation was slow in wetland slurries under both aerobic and anoxic conditions, with over 91% of the parent compound remaining after 142 days. 5:3 acid was detected only in anoxic conditions. Microbial shift was observed: Methylocaldum was enriched aerobically, while Methanomethylovorans increased under anoxic conditions.	[63]
Landfill leachate (aerobic conditions)	750 μg L^–1^	PFBA, PFPeA, PFHxA	10 days	Microbial analysis suggests that Actinobacteria (specifically the genus *Arthrobacter*) are likely key contributors to the biotransformation and the resulting production of short-chain PFCAs. Biotransformation was significantly enhanced (increasing from ~7% to ~20% removal) under nitrification-induced conditions.	[61]
Landfill sediment and leachate (aerobic conditions)	650 μg L^–1^	PFHpA, PFOA, PFPeA, PFBA, PFHxA	60 days	Biotransformation in landfill sediments was substrate-dependent, with higher removal (~21%) observed in leachate-rich microcosms compared to deionized water (~14%). Proteobacteria dominated the microbial community; Acidobacteria and Actinobacteria increased over 60 d, likely contributing to transformation processes.	[64]
Agricultural toposoil with earthworms (*Eisenia fetida*)	1213 nmol g^–1^ dry soil	TFA, PFBA, PFPrA, PFPeA, PFHxA	20 days (in vivo assays) and 32 h (in vitro trials)	Earthworms bioaccumulated 6:2 FTSA from soil and facilitated its biotransformation through α-oxidation pathway, with CYP450 and GST identified as the primary enzyme systems involved. TFA was the dominant terminal product, and PFHpA was not detected in earthworms or soil.	[50]
Bacteria culture (*Rhodococcus jostii* RHA1) in sulfur-rich and sulfur-free mineral salt media	20 mg L^–1^	6:2 FTUSA, 6:2 FTCA, 6:2 FTUCA, α-OH 5:3 FTCA, PFHpA	144 h	Biotransformation of 6:2 FTSA by *R. jostii* RHA1 was strictly modulated by sulfur availability, occurring only under S-free conditions where it served as the sole sulfur source. Desulfonation is the rate-limiting step that must precede defluorination. Key enzymes were identified: alkanesulfonate monooxygenase for desulfonation, and haloacid dehalogenase, alkane monooxygenase, and CYP450s for defluorination.	[58]
Sany soil with sulfur-rich and sulfur-limited nutrient solutions, incubated with *R. jostii* RHA1 and plants (*Arabidopsis thaliana* ecotype Col-0)	1.5 mg kg^–1^	6:2 FTUCA, PFPeA, PFHxA, PFHpA	25 days	Biodegradation in soil was primarily driven by sulfur-limiting conditions and was significantly enhanced through bioaugmentation with *R. jostii* RHA1 and the addition of 1-butanol as an enzyme inducer. *A. thaliana* accumulated 6:2 FTSA from the soil, with a limited impact on biodegradation, potentially because sulfur-containing root exudates repress the microbial desulfonating enzymes.	[57]
Bacteria culture (*Dietzia aurantiaca* J3), with sulfur-free mineral medium	150 μM	6:2 FTCA, 6:2 FTUA, 5:3 FTCA, PFHxA, PFPeA	7 days	Strain J3, isolated from landfill leachate, completely degraded 6:2 FTSA by using it as a sole sulfur source. Proteomic analysis revealed upregulation of an alkanesulfonate monooxygenase gene cluster, indicating its role in 6:2 FTS uptake and desulfonation.	[54]
Aerobic sludge	1.2 mg L^–1^	6:2 FTUSA, Ketone-6:2 FTSA, OH-6:2 FTSA, 5:3 FTU amide, 5:3 FT amide-AcA, 5:2 FTUCA, 4:2 FTUCA, PFHpA, PFHxA, PFPeA, PFBA	100 days	6:2 FTSA had an estimated half-life of 28.8 days. Novel initial biotransformation pathways involving dehydrogenation and hydroxylation processes, and the desulfonation and oxidation of ketone-6:2 FTSA as a significant metabolic source for PFHpA (the most abundant biotransformation product).	[55]
Aerobic wetland soil slurry	150 μg L^–1^	6:2 FTUSA, Ketone-6:2 FTSA, 5:2 sFTOH, PFHpA, PFHxA, PFPeA, PFBA	100 days	6:2 FTSA underwent relatively rapid biotransformation with half-lives of 16.7–26.2 days. Dehydrogenation was identified as the primary initial step, and supplementation with sodium acetate accelerated degradation by enriching functional bacterial genera (Hydrogenophaga and Rhodococcus).	[65]
Fungal culture (*Trametopsis cervina*) in sulfur-rich medium	1.15 μg mL^–1^	5:2 sFTOH, 5:3 FTCA, PFHxA, PFBA, PFPeA	30 days	The white-rot fungus *T. cervina* transformed ~50 mol% of 6:2 FTS within 30 days. Degradation occurred despite sulfur-rich conditions, suggesting this species utilizes non-specific enzymes for desulfonation that are unrelated to sulfur-acquisition metabolism.	[53]
Bacteria culture (*Labrys portucalensis* F11)	10,000 μg L^–1^	4:2 FTS	100 days	*L. portucalensis* F11 caused a 21% decrease in 6:2 FTSA concentration over 100 days. Biodegradation was confirmed by a measured increase in fluoride ions (1% defluorination), demonstrating the strain’s ability to cleave stable C-F bonds.	[52]
Mediterranean and arid soils	10, 50 and 250 ng g^–1^ dry soil	PFHpA	210 days	6:2 FTSA breakdown was higher in Mediterranean soil than in arid soil, coupled with a higher occurrence of terminal PFHpA. Alkaline phosphomonoesterase and phosphodiesterase activities suggested as sensitive indicators for soil monitoring of 6:2 FTSA contamination.	This study

*PFPeA* perfluoropentanoic acid, *PFHxA* perfluorohexanoic acid, *PFBA* perfluorobutanoic acid, *6:2 FTUCA* 6:2 unsaturated fluorotelomer acid, *5:3 Uacid* 5:3 unsaturated fluorotelomer acid, *5:3 acid* 5:3 fluorotelomer acid, *6:2 FTCA* 6:2 fluorotelomer carboxylic acid, *6:2 FTUA* 6:2 fluorotelomer unsaturated acid, *5:3 FTCA* 5:3 fluorotelomer carboxylic acid, *5:2 FT ketone* 5:2 fluorotelomer ketone, *5:2 sFTOH* 5:2 fluorotelomer secondary alcohol, *6:2 FTOH* 6:2 fluorotelomer alcohol, *PFPrA* perfluoropropionic acid, *TFA* trifluoroacetic acid, *GST* glutathione *S*-transferase, *CYP450s* cytochrome P450 enzymes, *α-OH 5:3 FTCA* α-hydroxy-5:3 fluorotelomer carboxylate, *ketone-6:2 FTSA* ketone-substituted 6:2 fluorotelomer sulfonic acid, *OH-6:2 FTSA* hydroxyl-substituted 6:2 fluorotelomer sulfonic acid, *5:3 FTU amide* 5:3 fluorotelomer unsaturated amide, *5:3 FT amide-AcA* 5:3 fluorotelomer amide acetic acid.

The contrasting behavior of PFOA and 6:2 FTSA further highlights compound-specific PFAS fate in soil. PFOA remained stable over 210 days in both soils, whereas 6:2 FTSA dissipated in both soils and did so more strongly in the Mediterranean soil. This stronger dissipation may reflect not only abiotic soil differences but also the generally higher microbial activity indicators observed in that soil, although the mechanisms responsible for fluorotelomer transformation were not resolved here. More broadly, the soil-dependent differences in PFAS effects and dissipation should be interpreted as responses emerging from multiple soil properties rather than from TOC alone.

These findings also need to be viewed in the context of real agricultural exposure pathways. In this study, PFAS were directly spiked into soils, whereas agricultural inputs more often occur through biosolids, sewage sludge, or urban compost. Such amendments may initially reduce PFAS bioavailability by increasing sorption to organic matter, but they can also promote PFAS mobilization through dissolved organic matter, rhizosphere-associated desorption, and precursor biotransformation^20,21,63^. Recent studies further indicate that biosolid-amended soils may contain hidden precursor pools and treatment-dependent microbial responses that alter both PFAS retention and biological exposure over time^15^. Our results therefore support a precautionary approach to repeated application of PFAS-contaminated amendments, particularly in dryland agricultural soils with limited sorptive buffering and greater biochemical sensitivity. Future work should test these patterns under field conditions and in amended soils, while integrating PFAS residues, precursor dynamics, and biochemical indicators into soil health monitoring frameworks.

## Methods

### Field study and soil sampling

Two agricultural soils were collected for this study: an Alfisol (Xeralf) from the experimental field station of the regional government of Castilla-La Mancha (39°52'04“N, 4°02'37“W, Toledo, Spain) and an Aridisol (Argid)^64^ from abandoned cropland in northern Fuerteventura island (28°36'26“N, 13°54'27“W, Canary Islands, Spain). The former represents a Mediterranean agricultural soil, whereas the latter represents a desertification-prone arid agricultural soil^65^. Surface soil samples (0–15 cm) were sieved to 2 mm before the experiments were initiated. Throughout the study, these soils are referred to as the “Mediterranean” and “arid” soils.

### Experimental setup

Soils were spiked with PFOA and 6:2 FTSA at nominal concentrations of 0, 10, 50, and 250 ng g^‒1^ dry soil. These concentrations were selected to span literature-reported PFAS exposure levels from typical to hotspot contamination, based on reported concentrations of PFOA and 6:2 FTSA in sewage sludge, biosolids, compost, and impacted soils (Supplementary Table S3). Standard PFAS solutions (250 and 2.5 µg ml^‒1^) were prepared in ethanol:H_2_O (50:50, v/v) and applied to 100 g of air-dried soils. After overnight ethanol evaporation, the treated soils were homogenized with a spatula and then mixed with 400 g of clean soil to achieve the nominal PFAS concentrations. Each treatment included four replicates (80 g dry soil per replicate) placed in 100-ml polypropylene vessels. Control soils received the same ethanol:H_2_O treatment and evaporation procedure as PFAS-spiked soils. Soil moisture was adjusted to 30% (w/w), corresponding to 53% and 64% of water-holding capacity for the Mediterranean (0.57 ± 0.01 g H_2_O g^‒1^ dry soil, mean ± SD) and arid soils (0.47 ± 0.02 g H_2_O g^‒1^ dry soil), respectively. Vessels were randomly placed in an incubator at 20 °C in the dark, and moisture was maintained gravimetrically. Subsamples (~5 g) were collected from each vessel at 3, 14, 60, and 210 days to assess short- and long-term impacts of PFAS contamination. Samples were stored at 4‒5 °C until required for enzyme measurements (within one week of collection).

### Soil enzyme activities

The potential activities of enzymes involved in carbon acquisition (EST and BG), phosphorus acquisition (ALP and PDE), and nitrogen acquisition (PRO and NAG) were measured in soil-water suspensions (1:25, w/v). Preparation of suspensions followed Sanchez-Hernandez et al.^29^, and a detailed description of enzyme assays is in the Supplementary materials. Microbial activity was further assessed through dehydrogenase and catalase activities, which require viable microbial cells. Dehydrogenase activity was measured following von Mersi and Schinner^66^, and catalase activity following Trasar-Cepeda et al.^67^. A detailed description of soil enzyme activities is provided in the Supplementary Information.

### Physicochemical properties of soils

At the end of the incubation period, soil subsamples were collected to determine pH, electrical conductivity (EC), TOC, extractable NO_3_^−^, NH_4_^+^, and inorganic phosphate (P_i_). Soil pH and EC were measured in soil-water suspensions (1:5, w/v), which were centrifuged (10,000 × *g* for 5 min) and filtered (0.22 µm, nylon) for P_i_ determination^68^, using a calibration curve made with NaH_2_PO_4_ (0–50 µg PO_4_^3−^ ml^–1^). Extractable NH_4_^+^ and NO_3_^−^ were measured colorimetrically in 2 M KCl extracts (1:10, w/v) after 30-min orbital agitation (25 rpm, 24 °C) and subsequent centrifugation (10,000 × *g* for 5 min). Ammonium was determined using the salicylate method^69^, and NO_3_^−^ using the Griess reagent with VCl_3_ as reductant^70^. Calibration curves used NH_4_Cl (0–10 µg NH_4_^+^ ml^–1^) and KNO_3_ (0–2 µg NO_3_^−^ ml^–1^). TOC was quantified by the dichromate redox method^71^, using a sucrose standard curve (0–16 mg C ml^–1^). Elemental composition and concentrations of potentially toxic metals were also measured in soils (Supplementary Table S4). Methods are described in the Supplementary Information.

### PFAS residue analysis

Residues of 32 targeted PFAS analytes, including PFOA, 6:2 FTSA, and potential transformation products, were determined following Semerád et al.^72^. Briefly, soil samples (2 g) were homogenized with 10 g of sea sand and subsequently extracted by pressurized liquid extraction with methanol (three cycles at 150 °C and 1500 psi). The resulting organic extract was concentrated under a stream of nitrogen and purified using Supelclean™ ENVI-Carb™ solid-phase extraction cartridges. Analytes were eluted with an acidified methanolic solution and further evaporated before instrumental analysis. Quantification was performed by liquid chromatography-tandem mass spectrometry operating in negative electrospray ionization mode and scheduled multiple reaction monitoring. Two transitions were monitored for each analyte, one for quantification and one for confirmation. The full list of target analytes and the corresponding quantification parameters for the validated method used in this study, including instrumental limits of quantification, linearity ranges, and method limits of quantification, is provided in Supplementary Table S2.

### Data analysis

Variations in basal enzyme activities in control soils were evaluated using two-way repeated-measures ANOVA, with soil type as the between-subject factor and sampling time (3, 14, 60, 210 days) as the within-subject factor. Greenhouse–Geisser corrected degrees of freedom and *p*-values were used for repeated-measures effects. When significant effects were detected, pairwise comparisons of estimated marginal means were performed with Holm correction for multiple testing. Correlation matrices (Supplementary Fig. S1) were constructed using Spearman’s rank correlation coefficient to assess monotonic relationships among enzyme activities and microbial activity indicators (dehydrogenase and catalase) during incubation. Correlations were calculated separately for each soil type using individual replicate values from control soils pooled across sampling times, and matrices were visualized using heatmaps and scatterplots to facilitate interpretation.

PFAS effects on extracellular enzyme activities were assessed using three complementary approaches. First, the T-SQI was calculated according to Mijangos et al.^28^. The index was specifically developed to evaluate the effects of external environmental factors, including pollution and fertilization, on soil functioning^73^. The index was calculated separately for each sampling time as:

1 $$T-{SQI}={10}^{\log m+\frac{{\sum }_{i=1}^{n}\left(\log {n}_{i}-\log m\right)-{\sum }_{i=1}^{n}\left|\log {n}_{i}-\log \bar{n}\right|}{n}}$$

where *m* is the mean enzyme activity in the control soil (set to 100%), *n*_*i*_ is the activity of each enzyme in PFAS-treated soils expressed as a percentage of the corresponding control, n is the number of enzymes included in the calculation, and *n̄* is the mean of *n*_*i*_ values across enzymes. T-SQI values greater than 100% indicate stimulation of soil biochemical functioning, whereas values lower than 100% indicate impairment. For each treatment group, mean T-SQI values were compared to 100% using two-tailed one-sample *t*-tests with Bonferroni correction.

Second, nutrient-acquiring enzyme activities were grouped following Ma et al.^74^ as: *C*‒acq = (EST + BG)/2, *N*‒acq = (NAG + PRO)/2, and *P*‒acq = (ALP + PDE)/2; where EST is esterase activity, BG is β-glucosidase, NAG is *N*-acetyl-β-D-glucosaminidase, PRO is protease, ALP is alkaline phosphomonoesterase, and PDE is phosphodiesterase. Effects of PFAS concentration on these grouped enzyme activities were evaluated using one-way ANOVA with Welch’s correction, followed by Games–Howell post hoc test.

Third, eco-enzymatic stoichiometry was used to explore whether PFAS contamination altered apparent microbial nutrient acquisition patterns. Vector length and vector angle were calculated from proportional enzyme activities^33,75^ as:

2 $${{Vector}\,L}=\sqrt{\left({x}^{2}+\,{y}^{2}\right)}$$

3 $${{Vector}\,A}=\arctan \left(\frac{y}{x}\right)\times \frac{180}{\pi }$$

where *x* = (EST + BG)/(EST + BG + PRO + NAG) and *y* = (EST + BG)/(EST + BG + ALP + PDE). As recommended by Puissant^76^, no log transformation was applied. Higher vector length values indicate stronger apparent C limitation relative to N and P, whereas vector angle values below 45° indicate N limitation and values above 45° indicate P limitation. In addition, the alternative threshold of 55° proposed by recent theoretical work was also considered^77^. Differences in vector length and angle among treatments were evaluated using one-way ANOVA with Welch’s correction followed by Games–Howell post hoc comparisons. Similarly, PFAS residue data were analyzed separately for each soil, PFAS, and sampling time. Residual concentrations were compared among nominal treatment levels (10, 50, and 250 ng g⁻¹) by using one-way ANOVA with Welch’s correction followed by Games–Howell post hoc comparisons. For datasets showing strong non-normality or zero-inflation, particularly for 6:2 FTSA residues at 210 days, differences among treatment levels were evaluated using the Kruskal–Wallis test followed by Dunn’s post hoc test with Holm correction for multiple comparisons. Comparisons of PFAS residues between day 0 and day 210 within the same treatment were evaluated using paired-samples Wilcoxon signed-rank tests (*p* < 0.05). All statistical analyses were performed using the JASP (version 0.97).

## Supplementary information

Supplementary Information

## Data Availability

The majority of the data supporting the findings of this study are included in this published article and the supplementary information. Raw data, including soil enzyme activities, PFAS concentrations and soil physicochemical properties, are available at the Open Science Framework repository at https://osf.io/7vpx6/overview.
