# Supplementary material for: Soil enzyme dynamics under PFAS contamination in arid and Mediterranean soils
Source: NPJ Emerg Contam. 2026 Aug 3;2(1):30. doi: 10.1038/s44454-026-00050-4 (PMC13433283; doi:10.1038/s44454-026-00050-4)
Supplement: Supplementary file 1 [file 44454_2026_50_MOESM1_ESM.pdf]

Supplementary materials

Soil enzyme dynamics under PFAS contamination in arid and Mediterranean soils

Juan C. Sanchez-Hernandez[1,*], Natividad I. Navarro Pacheco[1], Ximena Andrade Cares[1], Jaroslav Semerad[2,3], Tomas Cajthaml[2,3] and Mallavarapu Megharaj[4]

[1]Laboratory of Ecotoxicology, Institute of Environmental Sciences, University of Castilla-La Mancha, 45071 Toledo, Spain.

[2]Institute for Environmental Studies, Faculty of Science, Charles University, Benatská 2, CZ-128 01, Prague 2, Czech Republic.

[3]Institute of Microbiology of the Czech Academy of Sciences, Vídeňská 1083, CZ-142 20, Prague 4, Czech Republic.

[4]Global Centre for Environmental Remediation (GCER), School of Science, College of Engineering, Science and Environment, University of Newcastle, Callaghan NSW 2308, Australia.

Content

Table S1. Two-way repeated-measures ANOVA outcomes (Greenhouse-Geisser corrected) for enzyme activities in control (PFAS-free) soils.

Table S2. Target PFAS analytes included in the LC-MS/MS method and detection/quantification parameters.

Table S3. Concentrations of PFOA and 6:2 FTSA reported in the literature for organic amendments (sewage sludge, biosolids and compost) and soil treated with these materials.

Table S4. Concentration of major elements and heavy metals in the Mediterranean and arid soils.

Figure S1. Spearman's correlation matrix between enzyme activities of control (PFAS-free) Mediterranean (A) and arid (B) soils across the 210-d incubation period.

Figure S2. Mean concentrations of extractable NH_4^+-N, NO_3^--N, inorganic phosphate (P_i), and total organic carbon in the Mediterranean and arid soils.

Figure S3. Mean (± SEM, n=4) pH values and electrical conductivity (EC) of Mediterranean and arid soils after 210 days of incubation.

Methodology for enzyme activities, PFAS analysis, and elemental composition and potentially toxic element measurements.

Table S1. Two-way repeated-measures ANOVA outcomes (Greenhouse-Geisser corrected) for enzyme activities in control (PFAS-free) soils during the 210-day incubation period. Soil type was treated as the between-subject factor and sampling time as the within-subject factor.

Dependent variable[1]	Source of variation[2]	*F-value*	d.f.	*P*-value	*Partial* η^2
EST	Soil	94.8	(1, 6)	< 0.001	0.94
	Time	2.45	(1.50, 9.10)	0.146	0.29
	Soil × time	3.66	(1.50, 9.10)	0.076	0.38
ALP	Soil	25.4	(1, 6)	0.002	0.81
	Time	2.95	(1.28, 7.67)	0.122	0.33
	Soil × time	0.33	(1.28, 7.67)	0.632	0.05
BG	Soil	156	(1, 6)	< 0.001	0.96
	Time	5.04	(1.41, 8.50)	0.044	0.45
	Soil × time	7.64	(1.41, 8.50)	0.017	0.56
NAG	Soil	262	(1, 6)	< 0.001	0.98
	Time	27.7	(1.88, 11.3)	< 0.001	0.82
	Soil × time	11.1	(1.88, 11.3)	0.002	0.65
PRO	Soil	897	(1, 6)	< 0.001	0.99
	Time	7.40	(1.37, 8.21)	0.02	0.55
	Soil × time	5.69	(1.37, 8.21)	0.036	0.48
DEH	Soil	216	(1, 6)	< 0.001	0.97
	Time	81.0	(1.38, 8.26)	< 0.001	0.93
	Soil × time	25.7	(1.38, 8.26)	< 0.001	0.81
CAT	Soil	245	(1, 6)	< 0.001	0.97
	Time	417	(1.63, 9.77)	< 0.001	0.98
	Soil × time	54.0	(1.63, 9.77)	< 0.001	0.90
PDE	Soil	1330	(1, 6)	< 0.001	0.99
	Time	56.6	(1.75, 10.51)	< 0.001	0.90
	Soil × time	35.1	(1.75, 10.51)	< 0.001	0.85

[1]EST=esterase, BG=β-glucosidase, ALP=alkaline phosphomonoesterase, PDE=phosphodiesterase, NAG=*N*-acetyl-β-D-glucosaminidase, PRO=protease, DEH=dehydrogenase, CAT=catalase. [2]Categorical independent variables: time=3, 14, 60, and 210 days; soil type=Mediterranean and and arid soils.

Table S2. Target PFAS analytes included in the LC-MS/MS method and detection/quantification parameters of the validated method adopted in this study, adapted from Semerad et al.[1].

Compound	ILOQ (ng ml^{-1})	Linearity (ng ml^{-1})	MLOQ (ng g^{-1})
Perfluoro-1-butanoic acid (PFBA)	0.17	0.1-100	0.3
Perfluoro-1-pentanoic acid (PFPeA)	0.15	0.1-100	0.4
Perfluoro-1-hexanoic acid (PFHxA)	0.7	0.5-100	0.4
Perfluoro-1-heptanoic acid (PFHpA)	0.26	0.1-100	0.4
Perfluoro-1-octanoic acid (PFOA)	0.3	0.1-100	0.3
Perfluoro-1-nonanoic acid (PFNA)	0.09	0.1-100	0.3
Perfluoro-1-decanoic acid (PFDA)	0.08	0.1-100	0.3
Perfluoro-1-undecanoic acid (PFUdA)	0.07	0.1-100	0.1
Perfluoro-1-dodecanoic acid (PFDoA)	0.31	0.5-100	0.3
Perfluoro-1-tridecanoic acid (PFTrDA)	0.54	0.5-100	0.6
Perfluoro-1-tetradecanoic acid (PFTeDA)	0.41	0.25-100	0.6
Perfluoro-1-butanesulfonate (PFBS)	0.45	0.1-100	0.7
Perfluoro-1-hexanesulfonate (PFHxS)	0.17	0.1-100	1.6
Perfluoro-1-heptanesulfonate (PFHpS)	0.15	0.1-100	0.7
Perfluoro-1-octanesulfonate (PFOS)	0.15	0.1-100	0.2
n-methyl-perfluoro-1-butanesulfonamide (MeFBSA)	0.62	0.5-100	2.5
Perfluoro-1-octanesulfonamide (FOSA)	0.42	0.25-100	1.7
n-ethyl-perfluoro-1-octanesulfonamide (EtFOSA)	0.65	0.5-100	1.4
Fluorinated telomer acid 3:3 (3:3FTA)	0.7	0.5-100	9.6
Fluorinated telomer acid 5:3 (5:3 FTA)	0.7	0.5-100	4.4
Fluorinated telomer acid 7:3 (7:3 FTA)	0.7	0.5-100	4.2
Potassium 9-chlorohexandecafluoro-3-oxanonane-1-sulfonate (9-Cl-PF3ONS)	0.05	0.05-100	0.3
Potassium 11-chloroeicosafluoro-3-oxaundecane-1-sulfonate (11-Cl-PF3OUdS)	0.05	0.05-100	0.3
Sodium dodecafluoro-3H-4,8-dioxanonanoate (NaDONA)	0.1	0.1-100	0.7
2,3,3,3-tetrafluoro-2-(heptafluoropropoxy)propanoic acid (Gen-X)	0.04	0.05-100	0.4
Perfluoro-1-octanesulfonamidoacetate (FOSAA)	0.5	0.5-100	2.0
n-methyl-perfluoro-1-octanesulfonamidoacetate (MeFOSAA)	0.25	0.25-100	0.8
n-ethyl-perfluoro-1-octanesulfonamidoacetate (EtFOSAA)	0.25	0.25-100	1.9
4:2 Fluorinated telomer sulfonic acid (4:2 FTSA)*	0.25	0.25-100	1.6
6:2 Fluorinated telomer sulfonic acid (6:2 FTSA)*	0.3	0.25-100	3.6
8:2 Fluorinated telomer sulfonic acid (8:2 FTSA)*	0.25	0.25-100	1.8
10:2 Fluorinated telomer sulfonic acid (10:2 FTSA)*	0.13	0.1-100	0.4

ILOQ=instrument limit of quantification, MLOQ=method limit of quantification. *Fluorotelomer sulfonic acids (FTSAs) are also referred to in the literature as fluorotelomer sulfonates (FTS).

Table S3. Concentrations of PFOA and 6:2 FTSA reported in the literature for organic amendments (sewage sludge, biosolids and compost) and soil treated with these materials.

Matrix	Material	PFOA	6:2 FTSA	Ref
Sewage sludge and WWTP solids	Domestic sludge, including primary, digested, and anaerobically digested sludge	Range: 5.2 to 29.4 ng g^{-1}.	Not measured	2
Sewage sludge and WWTP solids	Primary sludge and activated sludge from a municipal WWTP	Range: less than 6 to 12 ng g^{-1}. Mean (activated sludge): 6.7 ng g^{-1}.<br>Range: less than 6 to 8.2 ng g^{-1}.	Not measured	3
Sewage sludge and WWTP solids	Pooled WWTP solids from 19 Australian plants, including primary sludge, secondary sludge, lagoon sludge, and dredged lagoon solids	Mean: 2.6<br>Median less than LOQ. Range less than LOD to 25 ng g^{-1}.<br>Detection: 84%	Mean: 0.26 ng g^{-1}.<br>Range: less than LOD to 2.7 ng g^{-1}.<br>Detection: 26%	4
Sewage sludge	Sewage sludge from Hesse, Germany	Mean: 5.0 ng g^{-1}.<br>Maximum: 50.6 ng g^{-1}.<br>Range: less than LOQ to 50.6 ng g^{-1}.	Not measured	5
Biosolids	Archived U.S. biosolids from the 2001 National Sewage Sludge Survey	Mean: 34 ng g^{-1}.<br>Range: 11.8 to 70.3 ng g^{-1}.	Not measured	6
Biosolids	Municipal biosolids applied to agricultural fields in Illinois	Range: 8 to 68 ng g^{-1}.	Not measured	7
Biosolids	Final biosolids from 19 Australian wastewater treatment plants	Mean: 8.3 ng g^{-1}.<br>Median 4.9 ng g^{-1}.<br>Range: below reporting limit to 45 ng g^{-1}.<br>Detection: 100%	Mean 0.4 ng g^{-1}.<br>Range: non detect to 3.5 ng g^{-1}.<br>Detection: 35%	8

Matrix	Material	PFOA	6:2 FTSA	Ref
Biosolids	Final dewatered biosolids from 75 Australian wastewater treatment plants	Mean: 7.5 ng g^{-1}.<br>Median: 4.3 ng g^{-1}.<br>Detection: 83%	Mean: 7.8 ng g^{-1}.<br>Median: 1.5 ng g^{-1}.<br>Detection: 23%	9
Biosolids	Final treated solids from 190 Michigan wastewater treatment plants	Mean: 4.8 ng g^{-1}.<br>Median: 1.2 ng g^{-1}.<br>Maximum: 96 ng g^{-1}.<br>Detection: 63%	Mean: 9.8 ng g^{-1}.<br>Maximum: 2050 ng g^{-1}.<br>Detection: 17%	10
Biosolids	Post stabilized biosolids product from 27 WRRFs in the United States and Canada	One detection at 1.9 ng g^{-1}.<br>Detection: 3.7%	Not detected	11
Biosolids and sludge-derived fertiliser	Sludge fermented fertilizer intended for agricultural use in Japan	Mean: 11.1 ng g^{-1}.	Not resolved as 6:2 FTSA alone. Summed FTS concentration: 4.85 ng g^{-1}.	12
Compost	Commercial composts intended for agricultural land application in Quebec	Range: 1.1 to 4.0 ng g^{-1}.	Excluded from results because of blank contamination	13
Compost	Refined final compost from windrow and in vessel commercial composting facilities	Mean: 0.1 ng g^{-1}	Not detected	14
Compost	Commercial biosolid derived compost	Mean: 11.9 ng g^{-1}.	Mean: 4.22 ng g^{-1}.	15
Compost	Compost from the WAS 1 biosolids processing line	Mean: 19.6 ng g^{-1}.	Not reported	16
Amended soils	Biosolids-amended agricultural soils in Illinois	Approximately 1-35 ng g^{-1} in surface soil, depending on loading rate; detected to 120 cm depth	Not measured	7
Amended soils	Surface and vadose zone soils after long term biosolids application	Mean (surface soil): 11 ng g^{-1}.<br>Mean (substation): 3.7 to 32 ng g^{-1}.<br>Median (maximum): 16 ng g^{-1}.	Not measured	17

Matrix	Material	PFOA	6:2 FTSA	Ref
Amended soils	Soils from ten northeastern U.S. farms with paired control and treatment fields	Exact values reported were 2.7 ng g^{-1} (0–15 cm, depth) and 1.6 ng g^{-1} (15–30 cm).	Targeted but not quantified above reporting limits	18
Amended soils	Class A and Class B biosolids amended soils in a plant soil experiment	Range: 8.09 to 11.44 ng g^{-1} across treatment combinations	Not measured	19
Amended soils	Sludge applied soils near Decatur, Alabama	Maximum: 2500 ng g^{-1} (2007 survey)<br>Maximum: 320 ng g^{-1} (2009 survey	Not measured	20

Table S4. Concentration of major elements and heavy metals in the Mediterranean and arid soils used in this study.[a]

Variable	Mediterranean soil	Arid soil
Al (%)	2.10	4.81
Fe (%)	1.36	3.48
Ca (%)	4.15	0.88
K (%)	0.68	1.31
Mg (%)	0.67	0.79
As (mg/kg)	5.43	4.66
Cd (mg/kg)	1.49	0.59
Co (mg/kg)	5.51	14.2
Cr (mg/kg)	25.5	78.5
Cu (mg/kg)	27.1	18.4
Mn (mg/kg)	208.8	666.4
Mo (mg/kg)	0.12	0.05
Na (mg/kg)	332.1	1,280
Ni (mg/kg)	13.0	47.2
P (mg/kg)	2,385	356.6
Pb (mg/kg)	239.1	16.2
S (mg/kg)	313.4	192
Se (mg/kg)	1.83	4.34
Si (mg/kg)	440.0	226.1
Zn (mg/kg)	70.5	59.0

[a] External service analysis by Servicio Universitario de Instrumentación Científica (University of Murcia).

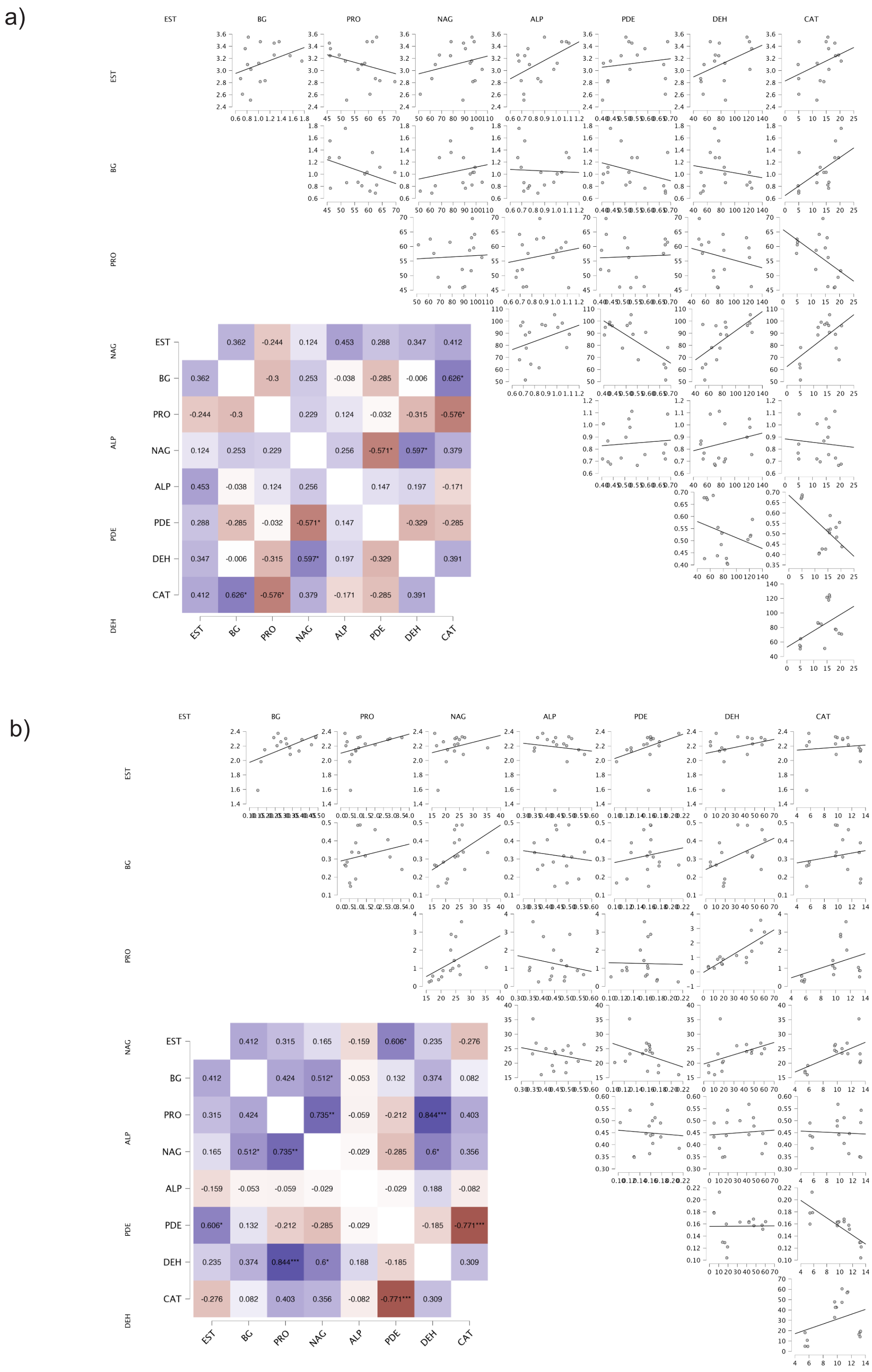

Figure S1. Spearman's correlation matrix of control soils across the 210-d incubation period. a) Correlation matrix coefficients and scatterplots for the Mediterranean soil. b) Correlation matrix coefficients and scatterplots.

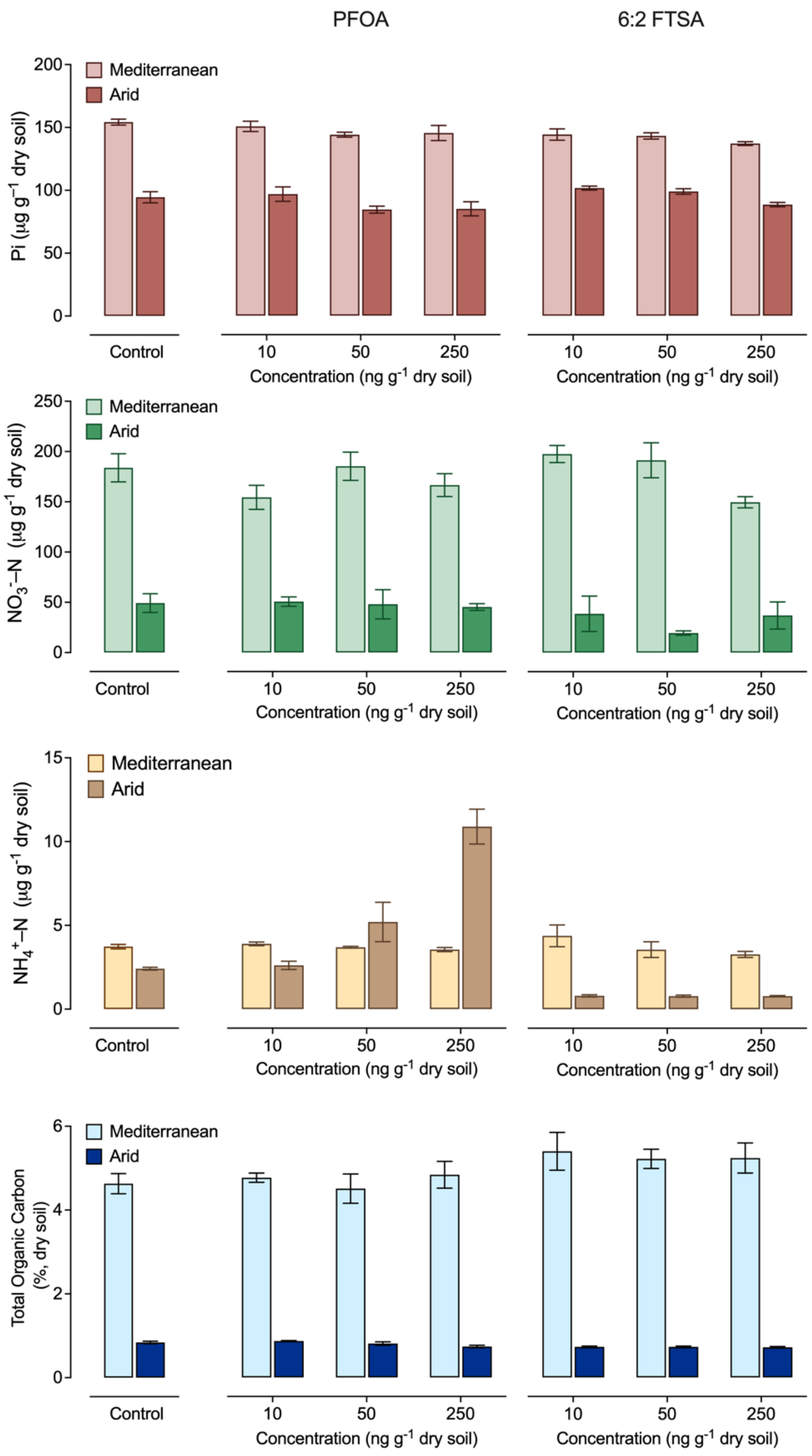

Figure S2. Inorganic nutrient concentrations and total organic carbon content. Mean (± SEM, n=4) concentrations of extractable NH_4^+-N, NO_3^--N, inorganic phosphate (P_i), and total organic carbon of Mediterranean and arid soils after 210 days of incubation with 0 (control), 10, 50, and 250 ng g^{-1} dry soil of PFOA and 6:2 FTSA.

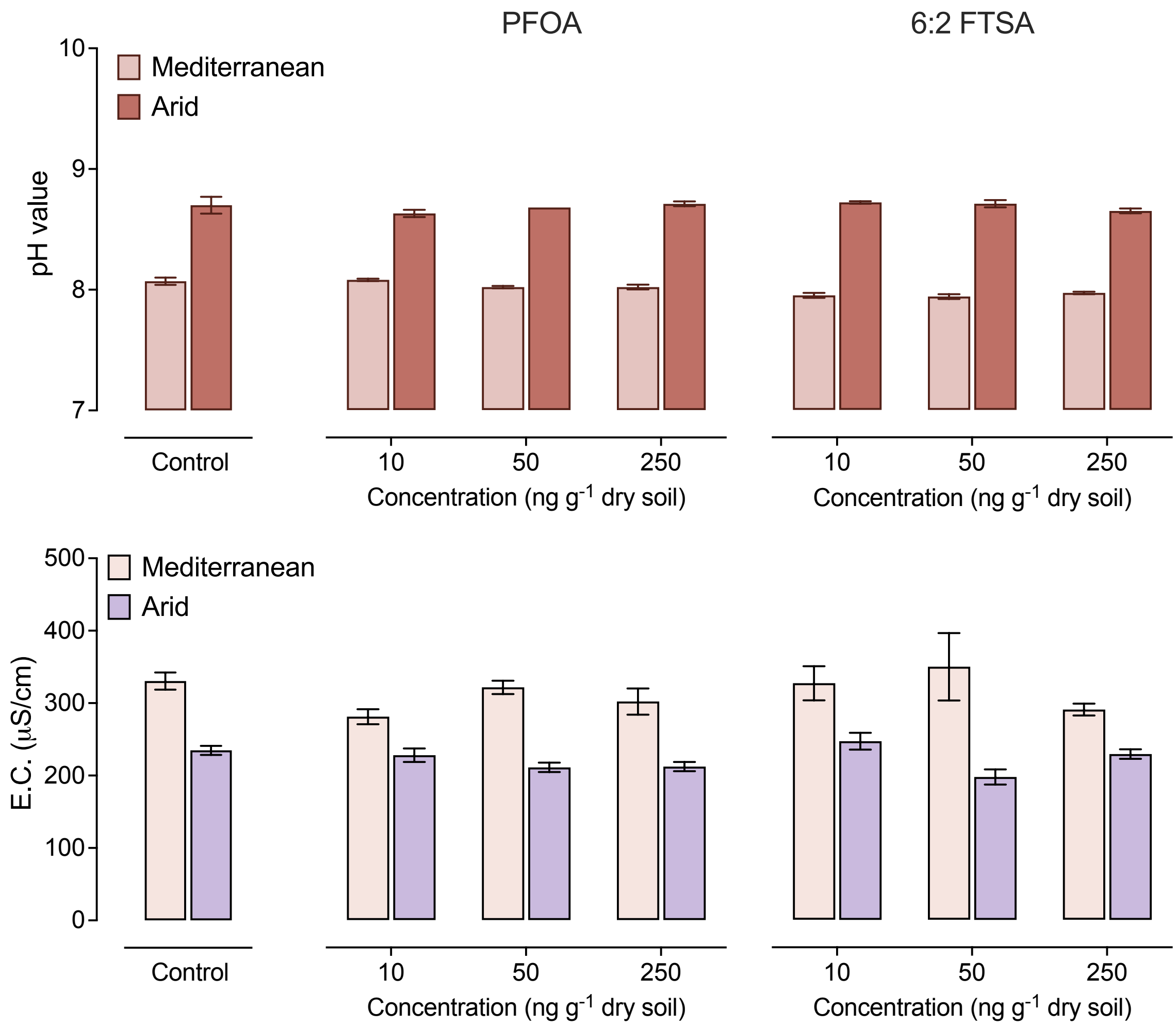

Figure S3. Soi-specific variations of pH values and electrical conductivity (EC). Mean (± SEM, n=4) pH values and EC of Mediterranean and arid soils after 210 days of incubation with 0 (control), 10, 50, and 250 ng g^{-1} dry soil of PFOA and 6:2 FTSA.

Methodology for enzyme activities, PFAS analysis, and elemental composition and potentially toxic element measurements

Soil enzyme activities

Esterase activity (EC 3.1.1.1)

Esterase (EST) activity was determined using 1-naphthyl butyrate (1-NB) as substrate in 96-well microplates. Reaction wells contained 380 µl of 0.1 M Tris-HCl buffer (pH=8.0), 100 µl of soil–water suspension, and 20 µL of 50 mM 1-NB. Microplates were incubated for 30 min at 25°C under continuous orbital shaking (Elmi® Skyline DTS-2, 800 rpm) and subsequently centrifuged at 2,500×*g* for 10 min at 10°C. Supernatants (150 µL) were transferred to clean microplates and mixed with 75 µL of a revealing solution composed of 0.1% Fast Red ITR dissolved in a 1:1 (v/v) mixture of 2.5% SDS and 2.5% Triton X-100. After 20 min of incubation in darkness, the absorbance of the 1-naphthol–Fast Red ITR complex was read at 530 nm. Activity was quantified against an external calibration curve prepared with 1-naphthol (1.5–100 nmol ml^{-1}) in the presence of soil–water suspension to correct for adsorption of the chromophore onto soil colloids. Results were expressed as µmol of product released h^{-1} g^{-1} dry soil.

Alkaline phosphomonoesterase (EC 3.1.3.2) and β-Glucosidase (EC 3.2.1.21) activities

Alkaline phosphomonoesterase (ALP) and β-glucosidase (BG) activities were measured following Popova and Deng[21] with minor adaptations. Reactions were assembled in microplate wells, each containing 200 µl of soil–water suspension, 200 µl of deionized water, and 100 µl of the corresponding substrate (4-nitrophenyl phosphate for ALP and 4-nitrophenyl β-D-glucopyranoside for BG) at a final concentration of 4 mM. Both substrates were dissolved in 20 mM modified universal buffer adjusted to pH=8.0. Microplates were incubated for 1 h at 25°C under continuous agitation, then centrifuged at 2,500×*g* for 10 min at 10 °C. Aliquots of 150 µl of supernatant were pipetted into new microplates and mixed with 75 µl of 0.5 M NaOH to terminate the reaction and develop the yellow colour of the hydrolytic product 4-nitrophenolate. Absorbance was read at 405 nm within 1 min of alkali addition. Enzyme activities were expressed as µmol h^{-1} g^{-1} dry soil using a 4-nitrophenol external calibration curve (5–100 nmol ml^{-1}).

Protease (EC 3.4.21.92)

Protease activity was quantified using casein as substrate, following Schinner et al.[22] with adaptations to a 2.0-ml microcentrifuge tube format. Each reaction consisted of 0.5 ml of soil–water suspension combined with 0.5 ml of a 2% (w/v) casein solution prepared in 50 mM Tris-HCl buffer (pH=8.1). Tubes were incubated under continuous agitation in a water bath at 50°C for 1h. Unreacted protein

was precipitated by the addition of cold 0.92 M trichloroacetic acid, followed by centrifugation at 10,000×*g* for 20 min at 4°C. Aliquots of 80 µl of the resulting supernatant were dispensed into microplate wells and treated with 120 µl of an alkaline copper reagent (50:1:1, v/v, 0.47 M Na_2CO_3 / 6 mM NaOH, 20 mM $CuSO_4$, and 26 mM sodium-potassium tartrate), followed by the addition of 80 µl of 10% (v/v) Folin–Ciocalteu's phenol reagent (Sigma-Aldrich, Madrid, Spain). The resulting blue complex was allowed to develop for 90 min in darkness prior to absorbance measurement at 700 nm. Protease activity was expressed as µmol tyrosine equivalents h^{-1} g^{-1} dry soil using an L-tyrosine external calibration curve.

Dehydrogenase activity (EC 1.1)

Dehydrogenase activity was determined according to von Mersi and Schinner[23], using iodonitrotetrazolium chloride (INT) as the terminal electron acceptor. For each assay, 1.0 g of soil was mixed with 1 ml of INT solution and 2.5 ml of 1 M Tris-HCl buffer (pH=7.0), then incubated for 60 min at 40°C in the dark under continuous agitation. The reaction was terminated by addition of 5 ml of a 1:1 (v/v) mixture of absolute ethanol and N,N-dimethylformamide. To ensure complete extraction of the reaction product, i.e., iodonitrotetrazolium formazan (INTF), tubes were maintained in the dark for a further 60 min with periodic agitation every 20 min. Following centrifugation at 4,500×*g* for 10 min, absorbance of the supernatant was measured at 464 nm. Dehydrogenase activity was expressed as µmol INTF h^{-1} g^{-1} dry soil, quantified against an external INTF calibration curve.

Catalase activity (EC 1.11.1.6)

Catalase activity was measured by an indirect spectrophotometric method based on Trasar-Cepeda et al.[24], in which residual hydrogen peroxide remaining after the enzymatic reaction is quantified colorimetrically. A soil–water suspension was first prepared by mixing 0.02 g of soil with 8 ml of deionized water for 30 min on a rotating mixer. The enzymatic reaction was initiated by adding 1 ml of 0.3% (w/v) H_2O_2 and incubating at room temperature (23-25°C) for 15 min, after which 1 ml of 3 M H_2SO_4 was added to stop the reaction. Tubes were centrifuged at 4,500×*g* for 10 min at 10°C, and the residual H_2O_2 in the supernatant was determined through an oxidative coupling reaction containing 110 mM Na-phosphate buffer (pH=7.4), 30 mM 4-aminoantipyrine, 5.3% (v/v) phenol, and 1.2 U ml^{-1} of horseradish peroxidase type I (5 KU; Sigma-Aldrich, Madrid, Spain). The dye product was read at 505 nm, and catalase activity was expressed as µmol H_2O_2 consumed h^{-1} g^{-1} dry soil, calculated from an H_2O_2 calibration curve.

N-acetyl-β-D-glucosaminidase activity (EC 3.2.1.52)

N-acetyl-β-D-glucosaminidase (NAG) activity was determined following Parham and Deng[25], with slight modifications for microplate format. Hydrolytic reactions were run in deep-well (700 µl) microplates. Aliquots (200 µl) of soil-water suspensions were incubated (continuous agitation) for 1h at 25ºC with 200 µl of 100 mM Na-acetate buffer (pH=5.5) and 100 µl of 10 mM p-nitrophenyl-*N*-acetyl-β-D-glucosaminide. Reactions were terminated by centrifugation (2,500 rpm, 5 min, 4ºC), and supernatants (150 µl) were transferred to flat-bottom 96-well microplates containing 75 µl of 0.5 M NaOH. The p-nitrophenolate formed was measured at 405 nm against blanks (sample-free). Non-enzymatic substrate decomposition was checked under the same assay conditions, and enzyme activity was corrected. Activities were expressed as µmol of 4-nitrophenolate h^{-1} g^{-1} dry soil using calibration curves (1.5 to 50 nmol ml^{-1}).

Phosphodiesterase activity (EC 3.1.4.1)

Phosphodiesterase (PDE) activity was determined according to the method of Acosta-Martínez and Tabatabai[26], adapted to a microplate format. Soil suspensions (200 µl) were incubated for 8 h with 200 µl of 50 mM Tris–HCl buffer (pH=8.0), 100 µl of 10 mM bis-nitrophenyl phosphate, and 1 mM sodium azide as microbial inhibitor. After incubation, reactions were terminated by centrifugation (2,500 rpm, 5 min, 4°C). A 150 µl aliquot of the supernatant was then transferred to microplate wells containing 100 µl of 0.1 M Tris–NaOH (pH=12.0), and absorbance at 405 nm was recorded. Activity was expressed as µmol h^{-1} g^{-1} dry soil using a 4-nitrophenol external calibration curve (5–100 nmol ml^{-1}).

In all kinetic assays, substrate, substrate-free controls and soil-free blanks were included under the sample conditions to correct for background absorbance and non-enzymatic substrate hydrolysis.

PFAS extraction and instrumental analysis.

PFAS residues in soil were extracted and analyzed following the validated method described by Semerád et al.[1,27], using a targeted analyte list of 32 PFAS, including the spiked compounds and screened potential transformation products relevant to the present study (Table S4). Briefly, soil samples were homogenized with sea sand and extracted by pressurized liquid extraction using methanol in an ASE 200 accelerated solvent extractor under the following conditions: 150ºC, 1500 psi, and three extraction cycles with 5 min static periods. Methanolic extracts were concentrated under a gentle nitrogen stream prior to cleanup. Extract cleanup was performed using Supelclean ENVI-Carb solid-phase extraction cartridges with 500 mg sorbent. A 1-ml aliquot of the extract was acidified with 10 µl formic acid, loaded onto the cartridge, and eluted with four successive 2.5-ml portions of

methanol:formic acid (99:1, v/v). The eluate was evaporated under nitrogen and adjusted to a final volume of 1 ml before instrumental analysis.

Chromatographic determination was carried out using a Shimadzu Nexera 2 liquid chromatograph coupled to a SCIEX 4500 tandem mass spectrometer equipped with an electrospray ionization source operated in negative mode. Separation was achieved on an XSelect CSH C18 column (75×2.1 mm, 2.5 µm) fitted with a guard column of the same particle size. The mobile phases were acetonitrile:formic acid 99.5:0.5 as phase A and water:acetonitrile:formic acid 79.5:20.0:0.5 as phase B. The elution program started at 80% A and was maintained for 0.5 min. Phase A was then decreased to 10% at 5 min and held until 12 min, followed by a further decrease to 5% at 15 min and to 0% at 16 min. This composition was maintained until 20 min, after which the system was returned to 80% A at 25 min and held until 30 min for column re-equilibration. Detection was performed in scheduled multiple reaction monitoring mode using two transitions per analyte, one for quantification and one for confirmation. The main ion source settings were a source temperature of 650°C and an ion spray voltage of −5500 V.

Analytical quality control followed the validated workflow described in Semerád et al. [7]. Quantification was based on external calibration. The corresponding instrumental limits of quantification, linearity ranges, and method limits of quantification for all 32 target analytes are provided in Table S2. A 50 ng ml^{-1} calibration standard was injected after every 20 analytical runs to monitor instrumental stability. Procedural blanks prepared with uncontaminated sea sand were included after every 10 samples, and solvent blanks were analyzed after every 20 injections. To minimize background contamination during sample handling, glass containers were avoided. PFHpA was not detected above its quantification limit in procedural blanks or unspiked control soils

Elemental composition and potentially toxic element measurements

Both analyses were performed at the Scientific Instrumentation Service (Área Científica y Técnica de Investigación, ACTI) of the University of Murcia (Spain) (https://www.um.es/web/acti/contenido/servicios/instrumentacion-cientifica/prestaciones). Briefly, soil samples were subjected to microwave-assisted digestion using the ultraWAVE system (Milestone), which is based on Single Reaction Chamber (SRC) technology. Samples were digested in concentrated nitric acid, and the resulting digests were made up to a final volume of 25 mL with ultrapure water. Elemental concentrations in the digested solutions were determined using two complementary plasma-based spectrometric techniques. Trace and minor elements (including Al, As, Fe, and Cu) were quantified by Inductively Coupled Plasma Mass Spectrometry (ICP-MS) using an Agilent 7900 instrument. Major elements (Na, K, Mg, Ca, P, S, and Si) were determined by Inductively

Coupled Plasma Optical Emission Spectrometry (ICP-OES) using an Agilent 5110 instrument. Results are summarized in Table S4.

References

1. Semerád, J. *et al.* Screening for 32 per- and polyfluoroalkyl substances (PFAS) including GenX in sludges from 43 WWTPs located in the Czech Republic - Evaluation of potential accumulation in vegetables after application of biosolids. *Chemosphere* **261**, 128018 (2020).
2. Higgins, C. P., Field, J. A., Criddle, C. S. & Luthy, R. G. Quantitative Determination of Perfluorochemicals in Sediments and Domestic Sludge. *Environ. Sci. Technol.* **39**, 3946–3956 (2005).
3. Schultz, M. M. *et al.* Fluorochemical Mass Flows in a Municipal Wastewater Treatment Facility. *Environ. Sci. Technol.* **40**, 7350–7357 (2006).
4. Coggan, T. L. *et al.* An investigation into per- and polyfluoroalkyl substances (PFAS) in nineteen Australian wastewater treatment plants (WWTPs). *Heliyon* **5**, e02316 (2019).
5. Stahl, T., Gassmann, M., Falk, S. & Brunn, H. Concentrations and Distribution Patterns of Perfluoroalkyl Acids in Sewage Sludge and in Biowaste in Hesse, Germany. *J. Agric. Food Chem.* **66**, 10147–10153 (2018).
6. Venkatesan, A. K. & Halden, R. U. National inventory of perfluoroalkyl substances in archived U.S. biosolids from the 2001 EPA National Sewage Sludge Survey. *Journal of Hazardous Materials* **252–253**, 413–418 (2013).
7. Sepulvado, J. G., Blaine, A. C., Hundal, L. S. & Higgins, C. P. Occurrence and Fate of Perfluorochemicals in Soil Following the Land Application of Municipal Biosolids. *Environ. Sci. Technol.* **45**, 8106–8112 (2011).
8. Moodie, D. *et al.* Legacy and emerging per- and polyfluoroalkyl substances (PFASs) in Australian biosolids. *Chemosphere* **270**, 129143 (2021).

9. Nguyen, H. T., Thai, P. K., Kaserzon, S. L., O'Brien, J. W. & Mueller, J. F. Nationwide occurrence and discharge mass load of per- and polyfluoroalkyl substances in effluent and biosolids: A snapshot from 75 wastewater treatment plants across Australia. *J. Hazard. Mater.* **470**, 134203 (2024).
10. Link, G. W., Reeves, D. M., Cassidy, D. P. & Coffin, E. S. Per- and polyfluoroalkyl substances (PFAS) in final treated solids (Biosolids) from 190 Michigan wastewater treatment plants. *J. Hazard. Mater.* **463**, 132734 (2024).
11. Oza, S. *et al.* Per- and polyfluoroalkyl substances in untreated and treated sludge/biosolids from 27 water resource recovery facilities across the United States and Canada. *Water Environ. Res.* **97**, e70039 (2025).
12. Njuki, N., Tanaka, S., Wenjiao, L. & Oluwoye, I. Per- and polyfluoroalkyl substances (PFASs) profile in sludge biosolid fertiliser intended for agricultural use in Japan. *J. Hazard. Mater.* **507**, 141728 (2026).
13. Saliu, T. D. *et al.* PFAS profiles in biosolids, composts, and chemical fertilizers intended for agricultural land application in Quebec (Canada). *J. Hazard. Mater.* **480**, 136170 (2024).
14. Khair Biek, S. *et al.* Fate of per- and polyfluoroalkyl substances through commercial composting facilities. *Biores. Technol.* **428**, 132485 (2025).
15. Huang, X., Arye, G., Zhang, W. & Ronen, A. Perfluoroalkyl and polyfluoroalkyl substances release from biosolid-derived compost. *ACS Omega* **11**, 10062–10072 (2026).
16. Rahman, A. *et al.* Understanding dynamics of PFAS in biosolids processed through composting, thermal drying and high temperature pyrolysis. *J. Water Process Eng.* **72**, 107508 (2025).
17. Johnson, G. R. PFAS in soil and groundwater following historical land application of biosolids. *Water Res.* **211**, 118035 (2022).
18. Oviedo-Vargas, D., Anton, J., Coleman-Kammula, S. & Qin, X. Quantification of PFAS in soils treated with biosolids in ten northeastern US farms. *Sci. Rep.* **15**, 5582 (2025).

19. Kharel, M., Tian, M., Vu, T., Miao, Y. & Zhang, W. PFAS in plant-biosolids-soil systems: Distribution, fractionation, and effects on soil microbial communities. *J. Hazard. Mater.* **497**, 139754 (2025).
20. Washington, J. W., Yoo, H., Ellington, J. J., Jenkins, T. M. & Libelo, E. L. Concentrations, distribution, and persistence of perfluoroalkylates in sludge-applied soils near decatur, Alabama, USA. *Environ. Sci. Technol.* **44**, 8390–8396 (2010).
21. Deng, S. & Popova, I. Carbohydrate Hydrolases. in *SSSA Book Series* (ed. Dick, R. P.) vol. 9 185–209 (Wiley, 2011).
22. Schinner, F., Öhlinger, R., Kandeler, E. & Margesin, R. *Methods in Soil Biology*. (Springer Berlin Heidelberg, Berlin, Heidelberg, 1996).
23. Von Mersi, W. & Schinner, F. An improved and accurate method for determining the dehydrogenase activity of soils with iodonitrotetrazolium chloride. *Biol. Fertil. Soils* **11**, 216–220 (1991).
24. Trasar-Cepeda, C., Camiña, F., Leirós, M. C. & Gil-Sotres, F. An improved method to measure catalase activity in soils. *Soil Biol. Biochem.* **31**, 483–485 (1999).
25. Parham, J. A. & Deng, S. P. Detection, quantification and characterization of β-glucosaminidase activity in soil. *Soil Biol. Biochem.* **32**, 1183–1190 (2000).
26. Acosta-Martínez, V. & Ali Tabatabai, M. Phosphorus Cycle Enzymes. in *SSSA Book Series* (ed. Dick, R. P.) vol. 9, 161–183 (Wiley, 2011).
27. Semerád, J. *et al.* The driving factors of per- and polyfluorinated alkyl substance (PFAS) accumulation in selected fish species: The influence of position in river continuum, fish feed composition, and pollutant properties. *Sci. Total Environ.* **816**, 151662 (2022).
